# Postprandial glycaemic response to berry nectars containing inverted sucrose

**DOI:** 10.1017/jns.2016.44

**Published:** 2017-01-26

**Authors:** Riitta Törrönen, Jarkko Hellström, Pirjo Mattila, Kyllikki Kilpi

**Affiliations:** 1 Department of Clinical Nutrition, Institute of Public Health and Clinical Nutrition, University of Eastern Finland, PO Box 1627, FI-70211 Kuopio, Finland; 2 New Business Opportunities, Natural Resources Institute Finland, Myllytie 1, FI-31600 Jokioinen, Finland; 3 Finnsugar Ltd, Sokeritehtaantie 20, FI-02460 Kantvik, Finland

**Keywords:** Berries, Inverted sucrose, Postprandial glucose, Insulin: Polyphenols, i.d., internal diameter, SGLT1, sodium glucose co-transporter 1

## Abstract

Sucrose is commonly used for sweetening berry products. During processing and storage of berry products containing added sucrose, sucrose is inverted to glucose and fructose. We have previously shown that postprandial glycaemic response induced by intact sucrose is attenuated when sucrose is consumed with berries rich in polyphenols. It is not known how inversion of sucrose affects glycaemic response. We investigated postprandial glycaemic and insulinaemic responses to blackcurrant (*Ribes nigrum*) and lingonberry (*Vaccinium vitis-idaea*) nectars and a reference drink (water) sweetened with glucose and fructose, representing completely inverted sucrose. The nectars and reference drink (300 ml) contained 17·5 g glucose and 17·5 g fructose. Polyphenol composition of the nectars was analysed. A total of eighteen healthy volunteers participated in a randomised, controlled, cross-over study. Blood samples were collected at fasting and six times postprandially during 120 min. Inverted sucrose in the reference drink induced glycaemic and insulinaemic responses similar to those previously observed for intact sucrose. In comparison with the reference, the blackcurrant nectar attenuated the early glycaemic response and improved glycaemic profile, and the lingonberry nectar reduced the insulinaemic response. The responses induced by inverted sucrose in the berry nectars are similar to those previously observed for berry nectars containing intact sucrose, suggesting that inversion has no major impact on glycaemic response to sucrose-sweetened berry products. The attenuated glycaemic response after the blackcurrant nectar may be explained by inhibition of intestinal absorption of glucose by blackcurrant anthocyanins.

Berries contain antioxidant vitamins, minerals and fibre, and they are unique as rich sources of flavonoids and other polyphenols. Berries and their flavonoids, especially anthocyanins, have been associated with reduced risk of CVD^(^^1^^–^^4^^)^, type 2 diabetes^(^^5^^–^^7^^)^, the metabolic syndrome^(^^8^^)^, inflammation^(^^9^^–^^11^^)^ and cognitive decline^(^^12^^,^^13^^)^, and they may also contribute to healthy ageing^(^^14^^)^ and weight maintenance^(^^15^^)^. Thus, the scientific evidence of health benefits is encouraging, and the Nordic Nutrition Recommendations^(^^16^^)^ advise to increase consumption of berries as part of a balanced healthy diet.

Due to low sugar:acid ratio and high content of polyphenols, sour and bitter taste is typical for many berries. Sugar, such as sucrose or high-fructose corn syrup, is commonly used to increase acceptability of berry products. Although sugar helps to increase berry consumption, it may compromise the nutritional and health benefits of berries, e.g. by inducing postprandial hyperglycaemia. Therefore, it is important to understand the metabolic consequences of sugar consumed with berries.

Our previous studies have shown that berries may improve postprandial glycaemic control in healthy subjects when consumed with sucrose. The post-meal fluctuation of blood glucose and insulin concentrations induced by sucrose was alleviated when sucrose was consumed with a mixture of four berries (bilberries, blackcurrants, cranberries and strawberries)^(^^17^^,^^18^^)^. In addition, blackcurrants and lingonberries, either as whole berries or nectars, improved the postprandial glycaemic and insulinaemic responses to sucrose^(^^19^^)^. These responses are consistent with delayed digestion of sucrose and/or slower absorption of glucose caused by berry polyphenols.

Sucrose is a disaccharide composed of glucose and fructose. In the presence of low pH and heat, sucrose is inverted, i.e. the glycosidic linkage is hydrolysed in an irreversible reaction, releasing glucose and fructose. In our previous studies^(^^17^^–^^19^^)^, sucrose was added to the berry products just before the product was consumed, to avoid inversion. However, during processing and storage of berry products with added sucrose, inversion of sucrose is likely to occur, due to the low pH of berries. Therefore, understanding the effects of berries on the glycaemic response induced by inverted sucrose is of practical importance. The purpose of this study was to investigate the postprandial glycaemic and insulinaemic responses to blackcurrant (*Ribes nigrum*) and lingonberry (*Vaccinium vitis-idaea*) nectars containing inverted sucrose, added as glucose and fructose, in healthy subjects.

## Methods

### Participants

Volunteers were recruited by an announcement in a local newspaper in the area of Kuopio, Finland. The inclusion criteria were as follows: healthy men or women, age 25–69 years, BMI 20–28 kg/m^2^, no diabetes or other chronic disease, no smoking, no antibiotic medication within the past 3 months, and no blood donation within the past month. At the screening visit, their health status and medical history was assessed by using a structured interview, and by anthropometric and laboratory measurements (routine haematological measurements, fasting plasma glucose, lipids, thyroid-stimulating hormone, alanine aminotransferase and creatinine).

The study was conducted according to the guidelines laid down in the Declaration of Helsinki and all procedures involving human subjects were approved by the Research Ethics Committee of the Hospital District of Northern Savo (Finland). Written informed consent was obtained from all participants. The study was registered at clinicaltrials.gov as NCT02743130.

### Test products

Blackcurrant and lingonberry nectars were prepared by Eckes-Granini Finland Oy Ab from industrial juice concentrates, with the final juice concentration of 50 %. Sodium benzoate was added as a preservative, and the nectars were pasteurised and stored at +5°C. Sugar composition was analysed by HPLC. In the research laboratory, glucose (*Glucosum anhydricum* Ph.Eur.; Vitabalans Oy) and fructose (Fruisana;, Danisco Sweeteners Oy) were added to achieve final concentrations of 17·5 g in 300 ml nectar (Table 1). According to European legislation, products obtained by adding water and sugars to concentrated fruit juice are defined as nectars^(^^20^^)^. A reference drink contained 17·5 g glucose and 17·5 g fructose in 300 ml tap water. Polyphenols (anthocyanins, proanthocyanidins, flavonols and phenolic acids) in the nectars were analysed before addition of the sugars.

**Table 1.** Sugar composition of the test products

	Reference drink	Blackcurrant nectar	Lingonberry nectar
Glucose, natural (g)		4·68	4·50
Glucose, added (g)	17·50	12·82	13·00
Fructose, natural (g)		5·07	3·81
Fructose, added (g)	17·50	12·43	13·69
Juice (ml)		300	300
Water (ml)	300		

Reference standards for anthocyanins (delphinidin-3-glucoside, delphinidin-3-rutinoside, cyanidin-3-galactoside, cyanidin-3-glucoside, cyanidin-3-arabinoside, cyanidin-3-rutinoside, petunidin-3-glucoside, peonidin-3-glucoside, malvidin-3-glucoside) were purchased from Extrasynthese. Triplicate samples were weighed and diluted with a mixture of methanol, acetic acid and water (65:4:31) prior to HPLC analysis. Anthocyanins were separated on a 150 mm, 4·6 mm internal diameter (i.d.), 5 µm, Gemini C18 column with a C18 guard column using a gradient elution of acetonitrile into 5 % formic acid according to Hellström *et al.*^(^^21^^)^. Detection wavelength was set at 518 nm and UV spectrum was recorded between 190 and 600 nm. Identification was based on reference standards, UV spectra and literature. Authentic standards were used for quantification when available; if not, the corresponding anthocyanidin-3-glucoside was used (e.g. petunidin-3-rutinoside was quantified as petunidin-3-glucoside).

Reference standards for proanthocyanidins (catechin, epicatechin and procyanidin B2) were purchased from Extrasynthese. Procyanidin oligomers (degree of polymerisation 3–10) were from Planta Analytica; the purity was determined with thiolysis^(^^22^^)^. Triplicate samples (10 ml) were purified with polyamide Supelco Discovery DPA-6S (Sigma-Aldrich Chemie Inc.)^(^^22^^)^. HPLC analyses were carried out with an Agilent 1100 serial device using a Phenomenex Luna HILIC column (250 mm, 4·6 mm i.d., 5 µm) with diol phase. The elution solvents were acetonitrile + acetic acid = 98 + 2 (A), methanol + acetic acid + water = 95 + 2 + 3 (B) and dimethylformamide + acetic acid + water = 85 + 2 + 13 (C). Proanthocyanidins were separated at the flow rate of 1 ml/min according to the following ternary gradient: 95 % A and 5 % B as initial conditions; changing to 40 % A and 60 % B in 45 min; isocratic elution (40 % A and 60 % B) for 5 min; changing to 60 % B and 40 % C in 1 min; changing to 20 % B and 80 % C in 2 min; isocratic elution (20 % B and 80 % C) for 7 min; changing to 80 % A and 20 % B in 4 min; changing to 95 % A and 5 % B in 1 min. Equalising time between the injections was 10 min. Fluorescence detection with excitation wavelength of 280 nm and emission wavelength 323 nm was used for the determination.

Flavonol glycosides were hydrolysed by refluxing the samples in 1·2 m-HCl in 50 % aqueous methanol for 1 h according to Hertog *et al.*^(^^23^^)^. Flavonol aglycones were identified and quantified using an Agilent 1100 Series HPLC equipped with a diode array detector. The analytical column was a Nova Pak C18 (150 mm, 3·9 mm i.d., 4 µm, Waters) protected with the manufacturer's precolumn. The mobile phase consisted of 0·05 m-phosphate buffer (A) at pH 2·4 and methanol (B) (5–60 % B in 50 min followed by 60–90 % B in 6 min). Isorhamnetin, kaempferol, myricetin and quercetin were quantified at 370 nm. For identification purposes, UV/visable spectra were recorded at 190–600 nm.

Reference standards for phenolic acids (caffeic acid, ferulic acid, *p*-coumaric acid, cinnamic acid) were purchased from Sigma-Aldrich. Triplicate samples were filtrated and analysed by Agilent 1290 Infinite UHPLC equipped with a diode array detector. Phenolic acids were separated at a flow rate of 0·4 ml/min on a 50 mm, 2·1 mm i.d., 1·8 µm, Zorbax Eclipse Plus C18 column using a gradient elution of acetonitrile (B) into 50 mm-phosphoric acid (pH 2·5, A): 95 % A and 5 % B as initial conditions; isocratic elution for 1·2 min; changing to 85 % A and 15 % B in 4·05 min; changing to 80 % A and 20 % B in 5·75 min; changing to 50 % A and 50 % B in 5 min; isocratic elution for 1·2 min; changing to 95 % A and 5 % B in 0·8 min. Detection wavelengths were 280 nm and 329 nm, and UV spectrum was recorded at 190–400 nm. Phenolic acids were identified according to their UV spectra and quantified as the corresponding aglyconic forms.

### Study design

The randomised, controlled, crossover study was carried out single-blinded for the laboratory personnel and analyses. Each subject participated in three 2 h tests on separate visits, at least 3 d apart. The test products were served in a randomised order.

The participants were instructed to keep their diet, body weight and living habits constant during the study, abstain from alcohol for 2 d before the test, and to refrain from intensive physical activity for 12 h before the test. The day before the study visit, they were advised not to consume any berries or berry products. In the evening before the visit, they were instructed to consume a meal of choice and repeat that meal before every visit. Body weight was measured at every visit.

The tests were carried out in the morning after a 10–12 h overnight fast. For collection of venous blood samples, an intravenous catheter (BD Venflon™ Pro 18GA, 1·3 × 32 mm; Beckton Dickinson) was inserted in an antecubital vein of the forearm. Baseline blood samples were drawn at fasting (0 min) and other samples at 15, 30, 45, 60, 90 and 120 min after starting to consume the test product. The samples for plasma glucose measurements were collected in citrate–fluoride tubes and for plasma insulin measurements in EDTA tubes kept on ice. Plasma was immediately separated by centrifugation at +4°C and stored at −70°C.

Glucose concentrations were analysed with the hexokinase method using Konelab System reagents and a Konelab 20 XTi clinical chemistry analyser (Thermo Fisher Scientific). The intra- and inter-assay CV were 2·7 and 1·8 %, respectively. Insulin was measured with a chemiluminometric immunoassay using Liaison® Insulin reagents (DiaSorin Inc.) and a DiaSorin Liaison® analyser (DiaSorin Deutchland GmbH). The intra- and inter-assay CV were 3·7 and 3·8 %, respectively.

### Calculations and statistical analyses

The data were analysed by using IBM SPSS Statistics for Windows, version 21.0 (IMB Corp.), and are expressed as means and standard deviations or standard errors, as indicated. Linear mixed-effects modelling was used to compare the effects of the test products. Histograms were used for checking the normality of model residuals. Values of *P* < 0·05 were considered to be statistically significant.

The statistical significance of the product × time interaction was tested in the mixed-model analysis using participant as a random factor and product × time and their main effects as fixed factors. When the product × time interaction was statistically significant, the differences between the reference drink and berry nectars at individual time points were tested by *post hoc* analysis with Sidak adjustment for multiple comparisons.

The maximum increases from the baseline concentrations were calculated by subtracting the fasting value from the highest value. GraphPad Prism 5.03 for Windows software (GraphPad Software, Inc.) was used for calculation of the glycaemic profile for each subject and test product according to Rosén *et al.*^(^^24^^)^, by dividing the time (min) during which the plasma glucose was above the fasting concentration with the incremental peak value (mmol/l). The incremental AUC were calculated by using GraphPad Prism software, ignoring the area below the baseline (0 min) concentration. The differences between the reference and berry nectars were tested using participant as a random factor and product as a fixed factor in the mixed-model analysis.

## Results

After screening twenty-six volunteers, thirteen female and five male participants were enrolled to the study, and they all completed the three visits. The average time between visits was 7·6 (range 3–17) d. The basic characteristics of the participants are presented in Table 2. The body weights remained stable throughout the study period. The mean body weights were 67·5 (sd 9·6), 67·6 (sd 9·5) and 67·3 (sd 9·3) kg for the reference, blackcurrant nectar and lingonberry nectar visits, respectively.

**Table 2.** Basic characteristics of the participants (*n* 13 female and *n* 5 male) (Mean values, standard deviations and ranges)

	Mean	sd	Range
Age (years)	59	7	40–68
Weight (kg)	66·9	9·4	53–86
BMI (kg/m^2^)	23·9	2·2	20–28
Fasting plasma glucose (mmol/l)	5·4	0·3	4·9–6·1
Fasting plasma total cholesterol (mmol/l)	5·3	0·8	3·9–7·1
Fasting plasma LDL-cholesterol (mmoll/l)	3·0	0·9	1·7–5·7
Fasting plasma HDL-cholesterol (mmol/l)	2·1	0·4	1·2–2·8
Fasting plasma TAG (mmol/l)	0·9	0·3	0·5–1·6

There were no differences in the times spent drinking the test products. The reference drink was ingested in 2:36 (sd 0:59), blackcurrant nectar in 2:37 (sd 1:04) and lingonberry nectar in 2:31 (sd 0:59) min:s.

### Glucose response

**Fig. 1.**
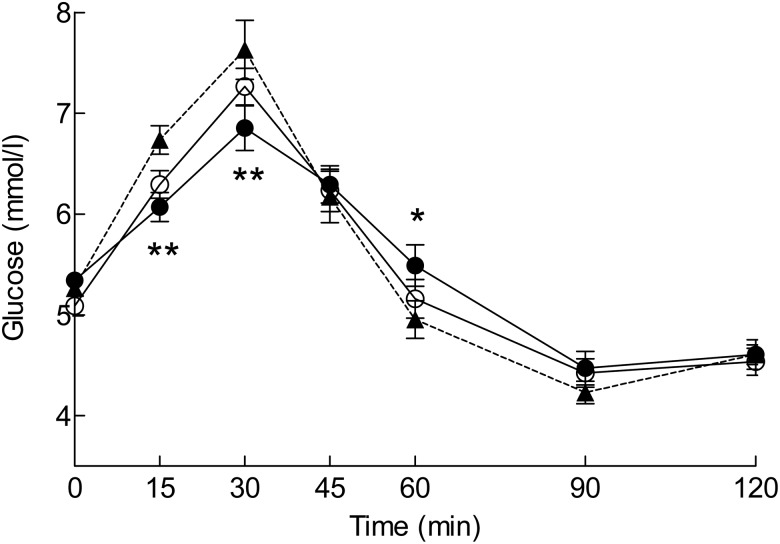
Plasma glucose concentrations after ingestion of the test products: reference (▲); blackcurrant nectar (●); lingonberry nectar (○). Values are means (*n* 17), with standard errors represented by vertical bars. *P* = 0·003 for product × time interaction in the mixed-model analysis. Mean value for the blackcurrant nectar was significantly different from that of the reference at an individual time point: * *P* < 0·05, ** *P* < 0·01 (*post hoc* analysis with Sidak adjustment).

Since one participant had an abnormal glucose response curve, the glucose variables have been calculated for seventeen participants. The postprandial glucose responses are presented in Fig. 1. The product × time interaction was statistically significant (*P* = 0·003). In comparison with the reference, ingestion of the blackcurrant nectar resulted in lower glucose concentrations at 15 and 30 min and a higher concentration at 60 min. After the blackcurrant nectar, the maximum increase from baseline was attenuated by 33 %, and the glycaemic profile was improved by 84 % (Table 3). The lingonberry nectar had no statistically significant effect on the glucose variables.

**Table 3.** Glucose and insulin variables after consumption of the test products (Mean values and standard deviations)

	Reference		Blackcurrant nectar		Lingonberry nectar		
	Mean	sd	Mean	sd	Mean	sd	Mixed model analysis: *P*
Glucose†							
Fasting (mmol/l)	5·3	0·3	5·3	0·3	5·1	0·4	>0·05
Maximum increase (mmol/l)	2·4	1·0	1·6**	0·9	2·2	0·5	0·007
AUC (mmol/l × min)	73·3	37·7	55·2	34·5	73·9	29·5	0·055
Glycaemic profile (min/(mmol/l))	25·2	7·2	46·4***	22·8	29·7	6·4	<0·001
Insulin‡							
Fasting (mU/l)	5·0	2·0	5·7	3·4	5·4	2·8	>0·05
Maximum increase (mU/l)	27·9	12·6	24·3	14·2	21·9*	12·1	0·040
AUC (mU/l × min)	1089	492	1117	482	958	436	0·090

Mean value was significantly different from that of the reference: * *P* < 0·05, ** *P* < 0·01, *** *P* < 0·001 (*post hoc* analysis with Sidak adjustment).

† *n* 17.

‡ *n* 18. To convert insulin in mU/l to pmol/l, multiply by 6·945.

### Insulin response

**Fig. 2.**
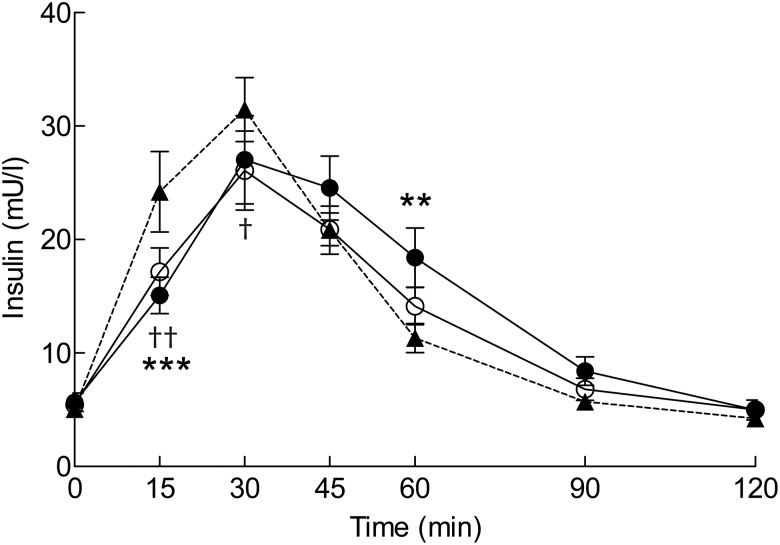
Plasma insulin concentrations after ingestion of the test products: reference (▲); blackcurrant nectar (●); lingonberry nectar (○). Values are means (*n* 18), with standard errors represented by vertical bars. *P* < 0·001 for product  ×  time interaction in the mixed-model analysis. Mean value for the blackcurrant nectar was significantly different from that of the reference at an individual time point: ** *P* < 0·01, *** *P* < 0·001 (*post hoc* analysis with Sidak adjustment). Mean value for the lingonberry nectar was significantly different from that of the reference at an individual time point: † *P* < 0·05, †† *P* < 0·01 (*post hoc* analysis with Sidak adjustment). To convert insulin in mU/l to pmol/l, multiply by 6·945.

The postprandial insulin responses are presented in Fig. 2. The product × time interaction was highly significant (*P* < 0·001). In comparison with the reference, lower insulin concentrations were observed at 15 min for both nectars and at 30 min for the lingonberry nectar. At 60 min, the insulin concentration remained higher after the blackcurrant nectar. In comparison with the reference, the maximum increase from the baseline was attenuated by 21 % after the lingonberry nectar (Table 3). After the blackcurrant nectar, the maximum increase from the baseline was 13 % smaller than after the reference, but the difference is not statistically significant.

### Polyphenol composition

Anthocyanins and proanthocyanidins were the major polyphenols in the nectars, and flavonols (quercetin) and phenolic acids (*p*-coumaric, caffeic and ferulic acids) were detected in smaller quantities (Table 4).

**Table 4.** Polyphenol contents in the nectars (mg/100 g) (Mean values and standard deviations of triplicate analyses)

	Blackcurrant nectar		Lingonberry nectar	
	Mean	sd	Mean	sd
Anthocyanins, total content	57·9	0·8	4·5	0·2
Delphinidin-3-glucoside	5·93	0·09	0·18	0·02
Delphinidin-3-rutinoside	28·5	0·40	–	
Delphinidin derivatives (as D-3-Glc)	–		0·84	0·05
Cyanidin-3-galactoside	–		2·19	0·09
Cyanidin-3-glucoside	2·63	0·04	0·45	0·02
Cyanidin-3-arabinoside	–		0·65	0·03
Cyanidin-3-rutinoside	19·5	0·30	–	
Petunidin-3-rutinoside	1·01	0·04	–	
Peonidin-3-rutinoside	0·33	0·03	–	
Malvidin-3-glucoside	–		0·17	0·01
Proanthocyanidins, total content	37·1	0·6	71·5	3·0
P1	0·50	0·014	3·43	0·19
P2	0·67	0·021	9·49	0·24
P3	0·49	0·039	7·53	0·22
P4–P6	1·26	0·13	15·2	0·6
P7–P10	0·72	0·061	6·78	0·65
>P10	33·5	0·5	29·1	1·4
Flavonols, total content	1·7	0·1	4·0	0·1
Quercetin	1·7	0·1	4·0	0·1
Phenolic acids, total content	1·72	0·02	1·17	0·01
Caffeic acid	0·741	0·011	0·24	0·001
Ferulic acid	0·22	0·002	0·17	0·001
*p*-Coumaric acid	0·76	0·010	0·37	0·006
Cinnamic acid	–		0·39	0·002

P, degree of polymerisation.

The content of anthocyanins was much higher in the blackcurrant nectar (57·9 mg/100 g) than in the lingonberry nectar (4·5 mg/100 g) (Table 4). The anthocyanin profile of the blackcurrant nectar was dominated by rutinosides of delphinidin and cyanidin followed by glucosides of the same anthocyanidins. Rutinosides of petunidin and peonidin were identified as minor anthocyanins. In the lingonberry nectar, cyanidin-3-galactoside was the most abundant, and cyanidin-3-arabinoside and cyanidin-3-glucoside were present in smaller amounts. In addition, traces of delphinidin and malvidin derivatives were detected, probably originating from contamination of the lingonberry juice concentrate with bilberry.

The lingonberry nectar contained more proanthocyanidins (71·5 mg/100 g) than the blackcurrant nectar (37·1 mg/100 g) (Table 4). Oligomeric proanthocyanidins (degree of polymerisation 2–10) dominated in the lingonberry nectar, and 35 % of the dimers were A-type (could not be estimated for other oligomers). On the other hand, 90 % of the proanthocyanidins in the blackcurrant nectar were highly polymerised (degree of polymerisation >10).

## Discussion

Sucrose is commonly used for sweetening berry juices and other berry products. When the metabolic effects of sucrose-sweetened berry products are evaluated, inversion of sucrose should be considered. In this study we investigated the effects of berry nectars sweetened with glucose and fructose, representing completely inverted sucrose, on postprandial glucose metabolism. Our results show that the early postprandial glycaemic responses induced by inverted sucrose in the berry nectars are similar to those observed in our previous studies^(^^18^^,^^19^^)^ where inversion of sucrose was avoided.

There are very few studies comparing the glycaemic effect of sucrose and inverted sucrose. The study of Ellwood *et al.*^(^^25^^)^ found no significant difference in the glycaemic response between a load of sucrose or inverted sucrose. Comparable glycaemic responses have also been reported for sucrose and high-fructose corn syrup, a sweetener consisting of free fructose and glucose^(^^26^^–^^29^^)^. However, it is possible that sucrose given in the form of a beverage in these studies was hydrolysed, as reported by Le *et al.*^(^^29^^)^. The present study shows that inverted sucrose induces postprandial glucose and insulin responses comparable with those of intact sucrose. The response curves of the reference drink containing 17·5 g glucose and 17·5 g fructose are practically similar to those reported previously for 35 g sucrose^(^^18^^,^^19^^)^. The maximum increases from the baseline were 2·4 and 2·3 mmol/l for glucose and 27·9 and 33·4 mU/l for insulin in this study and in the previous study^(^^19^^)^, respectively. The values for the glycaemic profile in these studies were 25·2 and 29·6 min/(mmol/l), respectively.

Blackcurrant in the form of a nectar (50 % juice) significantly attenuated the inverted sucrose-induced postmeal rise of plasma glucose concentration and improved the glycaemic profile, whereas the effect on insulin response was less pronounced. The lower glycaemic response was evident during the early postprandial phase, and in the later phase the declines of the glucose and insulin concentrations were less steep than after the reference. These results are identical to those detected previously for sucrose added to a similar blackcurrant nectar^(^^19^^)^. The maximum increase of glucose concentration from the baseline (1·6 mmol/l for both) and the values for the glycaemic profile (46·4 and 46·1 min/(mmol/l)) were similar in this study and in the previous study.

The glycaemic response induced by inverted sucrose in the lingonberry nectar did not differ from the reference. Also in the previous study^(^^19^^)^, sucrose in the lingonberry nectar had no effect on the early glucose response. However, intact sucrose in the lingonberry nectar produced a more sustained later-phase glucose response, prevented the sucrose-induced late postprandial hypoglycaemia and thereby improved the glycaemic profile^(^^19^^)^. These effects were not observed in the present study. Further studies are needed to verify and explain this difference. Inverted sucrose in the lingonberry nectar significantly decreased the early insulin response. Attenuation of postprandial insulin response by lingonberries, with little or no effect on glucose response, was previously observed when whole-berry purée was consumed with sucrose or white wheat bread^(^^19^^,^^30^^)^.

We have previously suggested that berries may modulate the postprandial glycaemic response to sucrose by reducing digestion of sucrose and/or absorption of glucose, and that polyphenols may be the active compounds^(^^17^^–^^19^^)^. In the small intestine, sucrose is hydrolysed to glucose and fructose by α-glucosidase, which is known to be inhibited by berry extracts and polyphenols *in vitro*^(^^31^^–^^33^^)^. In the present study, however, α-glucosidase activity plays no role, because sugar was ingested as free glucose and fructose. Therefore, the lower glycaemic response observed after consumption of the blackcurrant nectar cannot be explained by inhibition of α-glucosidase activity.

Inhibition of glucose absorption from the small intestine is another potential mechanism of glycaemic control after a meal. The intestinal absorption of glucose is mainly mediated by two transporters, the sodium glucose co-transporter 1 (SGLT1) in the apical membrane and GLUT2 in the basolateral membrane of enterocytes^(^^34^^,^^35^^)^. Polyphenols and polyphenol-rich extracts are known to interact with these transporters and may thereby regulate the rate of glucose absorption^(^^36^^,^^37^^)^.

In accordance with previous studies^(^^38^^–^^41^^)^, the polyphenol profile of the blackcurrant nectar was characterised by the abundance of anthocyanins, especially by rutinosides and glucosides of delphinidin and cyanidin, and highly polymerised proanthocyanidins, while lingonberry nectar was characterised by oligomeric and polymeric proanthocyanidins, with lower contents of anthocyanins and flavonols. Our previous^(^^19^^)^ and present results show that anthocyanin-rich blackcurrant is more potent than lingonberry in lowering post-meal glycaemia produced by intact or inverted sucrose, respectively.

There are no previous studies on the interaction of blackcurrant or its components with glucose absorption. However, *in vitro* studies have demonstrated that other anthocyanin-rich berries are able to influence intestinal glucose transport. In human intestinal Caco-2 cells, strawberry extract inhibited glucose uptake from the apical side into the cells and the GLUT2-facilitated efflux on the basolateral side^(^^42^^)^. Pelargonidin-3-glucoside, the main anthocyanin in strawberry, was responsible for 26 % of the total inhibition. Moreover, anthocyanin-rich berry extract decreased both Na-dependent and Na-independent glucose uptake and reduced SGLT1 mRNA and GLUT2 mRNA expression in Caco-2 cells^(^^43^^)^. Interestingly, cyanidin-3-glucoside and cyanidin-3-rutinoside were found to inhibit both types of glucose transport. These compounds and delphinidin glycosides were the main anthocyanins of the blackcurrant nectar of our study. A maqui berry extract rich in delphinidin glycosides and other anthocyanins decreased postprandial glucose and insulin concentrations after starch consumption, and SGLT1 inhibition by delphinidin was suggested to be the primary mechanism^(^^44^^)^.

In conclusion, the postprandial glycaemic and insulinaemic responses induced by inverted sucrose are similar to those observed previously for intact sucrose. Modulation of the early glycaemic response by berry nectars is practically similar for intact and inverted sucrose, suggesting that inversion has no major impact on glycaemic response to sucrose-sweetened berry products. The attenuated glycaemic response to the blackcurrant nectar containing inverted sucrose may be explained by inhibition of intestinal absorption of glucose by blackcurrant anthocyanins. In future studies, the effects of other anthocyanin-rich berries, such as blueberries, bilberries and chokeberries, on sucrose-induced postprandial glycaemia should be investigated.
